# Unveiling the distinctive mechanical and thermal properties of γ-GeSe

**DOI:** 10.1186/s40580-024-00436-3

**Published:** 2024-07-15

**Authors:** Jinsub Park, Yugyeong Je, Joonho Kim, Je Myoung Park, Joong-Eon Jung, Hyeonsik Cheong, Sang Wook Lee, Kwanpyo Kim

**Affiliations:** 1 Department of Physics, Yonsei University, Seoul, 03722 Republic of Korea; 2 Department of Physics, Ewha Womans University, Seoul, 03760 Republic of Korea; 3 Department of Physics, Sogang University, Seoul, 04107 Republic of Korea

**Keywords:** Young’s modulus, Thermal conductivity, Group-IV Monochalcogenide, γ-GeSe, Freestanding structure

## Abstract

γ-GeSe is a newly identified polymorph among group-IV monochalcogenides, characterized by a distinctive interatomic bonding configuration. Despite its promising applications in electrical and thermal domains, the experimental verification of its mechanical and thermal properties remains unreported. Here, we experimentally characterize the in-plane Young’s modulus (*E*) and thermal conductivity ($\:\kappa\:$) of γ-GeSe. The mechanical vibrational modes of freestanding γ-GeSe flakes are measured using optical interferometry. Nano-indentation via atomic force microscopy is also conducted to induce mechanical deformation and to extract the *E*. Comparison with finite-element simulations reveals that the *E* is 97.3$\:\pm\:$7.5 GPa as determined by optical interferometry and 109.4$\:\pm\:$13.5 GPa as established through the nano-indentation method. Additionally, optothermal Raman spectroscopy reveals that γ-GeSe has a lattice thermal conductivity of 2.3 $\:\pm\:$ 0.4 Wm^−1^K^−1^ and a total thermal conductivity of 7.5 $\:\pm\:$ 0.4 Wm^−1^K^−1^ in the in-plane direction at room temperature. The notably high $\:E/\kappa\:$ ratio in γ-GeSe, compared to other layered materials, underscores its distinctive structural and dynamic characteristics.

**Supplementary Information:**

The online version contains supplementary material available at 10.1186/s40580-024-00436-3.

## Introduction

Van der Waals (vdW) layered crystals have emerged as a promising material platform for exploring new phenomena and attaining a range of functionalities [1–3]. These crystals can be isolated down to a single-atomic-thickness unit by top-down exfoliation techniques or bottom-up synthesis, opening up various new avenues for tuning materials properties [4–7]. Moreover, these crystals exhibit a variety of polymorphic structures, with the inter-atomic arrangements of these polymorphs dictating the fundamental physical properties of the system being studied. For example, transition metal dichalcogenides (TMDCs) exhibit various polymorphs with distinct properties such as 2H, 1T, and distorted 1T (or 1T′) configurations [8–11]. An in-depth understanding of the formation of various polymorphic configurations and control of their phases can lead to new device platforms and applications [12–15]. 

Group-IV monochalcogenides are emerging as promising vdW materials, particularly noted for their potential in thermoelectric and phase change memory applications [16–22]. GeSe is a unique group-IV monochalcogenide, which exhibits various types of stable polymorphs at room temperature [23–26]. Recently, γ-GeSe has been recognized as a stable monochalcogenide featuring a distinctive intralayer structure, characterized by a Se-Ge-Ge-Se quadruple atomic sequence [24, 27–29]. Intriguingly, its inter-atomic bonding configuration deviates from the conventional 8*-N* rule, resembling instead the bonding pattern found in crystals exhibiting so-called metavalent bonding [27, 30]. Recent theoretical calculations have disclosed the low thermal conductivity ($\:\kappa\:$) of γ-GeSe, underscoring the crucial influence of its unique bonding configuration on this aspect of the material [31–35]. However, a comprehensive experimental investigation into the mechanical and thermal properties linked to this distinctive bonding configuration in γ-GeSe remains unreported. The experimental confirmation of γ-GeSe’s mechanical and thermal properties could pave the way for enhanced design and synthesis strategies, crucial for advancing thermoelectric and phase change memory technologies [27, 31, 33, 36–38]. 

In this study, we investigate the mechanical and thermal properties of γ-GeSe. By utilizing optical interferometry and eigenfrequency simulation through finite element method (FEM), we determined the mechanical resonance frequency and established the in-plane Young’s modulus (*E*) of γ-GeSe. We also derived the *E* employing nano-indentation via atomic force microscopy (AFM) and compared these findings with the results of bending simulations conducted using the FEM method. The experimentally measured *E* of γ-GeSe is consistent with the density functional theory (DFT) calculations reported in the literature. Moreover, using optothermal Raman spectroscopy and heat transfer simulations, we measured the lattice thermal conductivity ($\:{\kappa\:}_{\text{L}}$) and the total thermal conductivity ($\:{\kappa\:}_{\text{t}\text{o}\text{t}\text{a}\text{l}}$). Our findings verify that γ-GeSe possesses exceptional mechanical rigidity and comparatively low thermal conductivity among group-IV monochalcogenides, emphasizing the significance of its unique intralayer bonding structure.

## Results and discussion

**Fig. 1:**
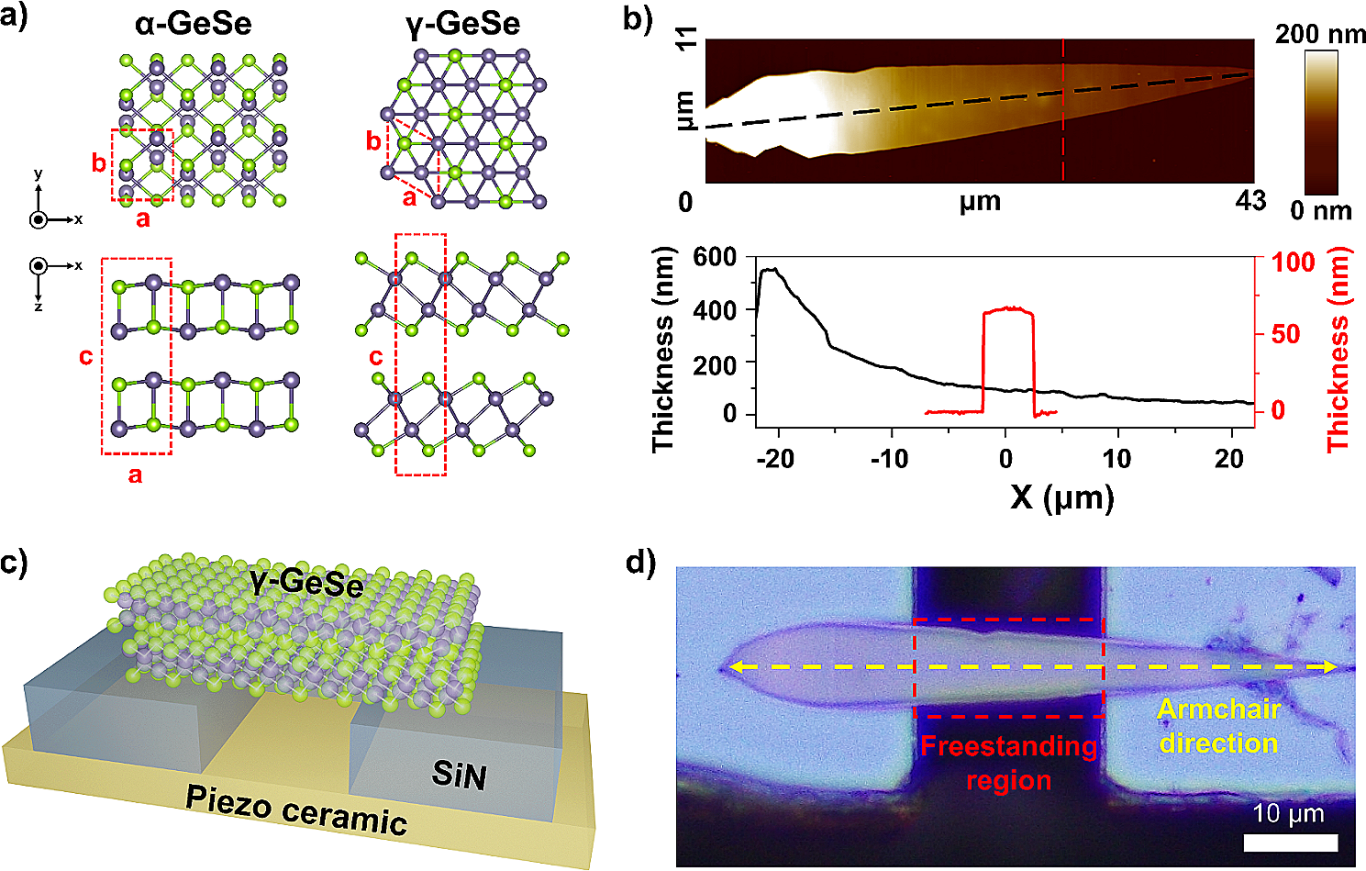
Fabrication process of the doubly-clamped freestanding γ-GeSe sample. **(a)** Crystal structures of α-GeSe and γ-GeSe. **(b)** AFM topography of a dagger-shaped γ-GeSe flake. The lower panel shows the height profiles of the flake along the dashed black and red lines. **(c)** Schematic of the doubly-clamped freestanding γ-GeSe sample. **(d)** Optical image of the fabricated freestanding γ-GeSe sample

Among the various polymorphic forms, α-GeSe is the most prevalent polymorph, exhibiting an orthorhombic layered-structure with a covalently bonded intralayer arrangement (Fig. 1a**)**. The anisotropic in-plane structure of α-GeSe leads to distinct physical properties along its zigzag and armchair lattice directions, including variations in electrical, thermal transport, and elastic characteristics [39–41]. Conversely, γ-GeSe features a distinct hexagonal crystal structure, characterized by individual layers that are four-atom-thick (Fig. 1a), as verified recently [24, 29]. The hexagonal symmetry of γ-GeSe suggests that it should exhibit isotropic physical properties within its plane. Moreover, in γ-GeSe, Ge exhibits an octahedral bonding characteristic, showing similarities to Te-based monochalcogenides, such as GeTe [27]. Te-based monochalcogenides are characterized by high anharmonicity and a unique electronic state known as metavalent bonding, [42] which renders them suitable for applications in thermoelectrics and phase-change memory.

Freestanding samples have to be fabricated to accurately measure mechanical vibration and heat transfer. In this study, doubly-clamped freestanding samples were prepared through dry-transferring to a Si_3_N_4_ trench substrate with a trench length of 18–25 μm. γ-GeSe flakes were synthesized using chemical vapor deposition (CVD) with an Au catalyst [24]. The samples prepared by this method usually exhibit a dagger shape tens of micrometers in size (Fig. 1b**)**. The one-sided nucleation edge of the samples shows a considerably thicker height (over 500 nm) compared to the comparatively uniform thickness (50–80 nm) of the main sample body. The relatively flat region with tens of micrometers in length can be used for the fabrication of freestanding samples (Fig. 1c and d**)**. Previous studies have confirmed that synthesized γ-GeSe typically exhibits an armchair lattice direction aligned with the dagger-shaped flake structure of the flake (Fig. 1d) [24]. To enhance the mechanical and thermal contact with the transferred γ-GeSe flake, we also deposited Au on both clamping sides (**Supporting Figure ****S1**). The individual freestanding samples were investigated using an optical microscope, scanning electron microscopy (SEM), and AFM to characterize their thickness profile, width profile, and length (**Supporting Figure S2**). The thickness profile of the γ-GeSe flakes measured by SEM from a side-view was consistent with those measured by AFM. The thickness and width of the freestanding region were found to be within the ranges of 60–420 nm and 5–10 μm, respectively.

**Fig. 2:**
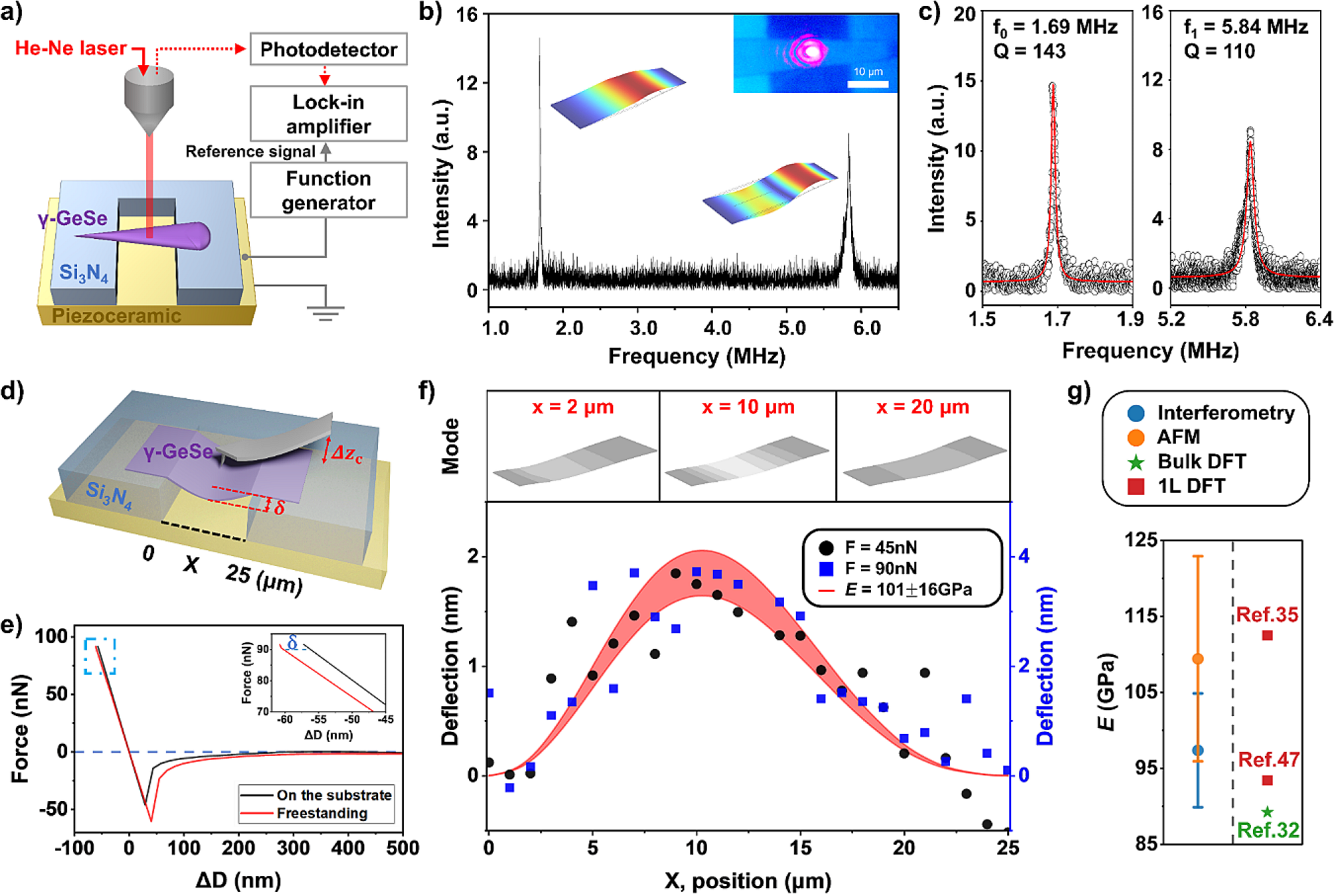
Young’s modulus measurement of γ-GeSe based on optical interferometry and nano-indentation. (**a**) Schematic of the optical interferometry measurement setup. (**b**) Measured optical intensity as a functional of driving frequency of the piezo ceramic substrate. The fundamental and second resonance modes are denoted by dashed rectangles. The upper right inset shows an optical image during the measurement. (**c**) Zoomed-in optical intensity spectra for vibration modes of the fundamental (left) and second resonance (right). (**d**) Schematic of the nano-indentation measurement based on AFM. (**e**) Force–distance curves on the substrate (black) and the freestanding region (red), respectively. The inset shows the different slopes in the zoomed-in force–distance curves from the dashed rectangle. (**f**) Sample deflection as a function of position in the suspended region. The red line indicates the standard deviation with *n* = 20. **(g**) Experimentally measured Young’s modulus of γ-GeSe via optical interferometry and indentation method. The values from theoretical calculations are shown for comparison [33, 37, 50]

To explore the vibrational properties of γ-GeSe, we employed optical interferometry measurements, a method extensively used for investigating the mechanical properties of various emerging materials [43–46]. A piezo-ceramic substrate was attached beneath the freestanding device to stimulate mechanical vibration. The vibration signal of the samples was optically detected using a lock-in amplifier (Fig. 2a**)**. Figure 2b shows a representative optical signal recorded as a function of excitation frequency. Significant resonance signals were detected near 1.7 MHz and 5.8 MHz (Fig. 2b and c**)**. Furthermore, FEM simulations were employed to investigate the mechanical properties of γ-GeSe. For these simulations, the identified geometrical shapes of the freestanding samples and their *E* parameters were used as inputs (**Supporting Note 1)**. We found that the measured mechanical resonances correspond to the fundamental and second vibrational modes of the doubly-clamped sample with *E* = 93 GPa. The simulated frequency ratio between these two peaks was found to be 3.43, consistent with the experimental frequency ratio of 3.46. The quality factor for each mode was measured to be 143 and 110, respectively (Fig. 2c).

Nano-indentation is another widely used method for measuring *E* of nano materials [46–49]. We conducted nano-indentation using AFM to study the bending properties of γ-GeSe (Fig. 2**)**. As the Z-scanner descends, the AFM tip approaches the sample, causing both the freestanding sample and the AFM cantilever to bend (Fig. 2d). During AFM operation, the movement of the Z-scanner ($\:\varDelta\:D$) results in the deflection of the AFM cantilever ($\:\varDelta\:{z}_{\text{c}}$) as well as the deflection of the sample ($\:\delta\:$). This relation can be expressed as

1 $$\:\varDelta\:D=\delta\:+\varDelta\:{z}_{\text{c}}$$

2 $$\:F=k\times\:\varDelta\:D$$

where the spring constant ($\:k$) of the AFM cantilever was calibrated by the built-in thermal tune method. For example, we acquired the force–distance curve of the γ-GeSe at the center position (X = 10 μm) in the suspended region and on the Si_3_N_4_ substrate (Fig. 2e). The sample deflection $\:\:\delta\:\:$on the hard substrate was assumed to be zero. The deflection of the sample was found to be 3.72 nm at the center position (X = 10 μm), calculated based on the difference between two force–distance curves.

To understand the bending properties of γ-GeSe more precisely, we acquired force–distance curves at different locations along the suspended sample at a distance interval of 1 μm by applying forces of 45 nN and 90 nN sequentially at each point. The deflection data at different locations is shown in Fig. 2f. Indentation measurements taken at various locations on the sample confirmed a higher degree of deflection near the central position. The deflection profiles obtained from the experiment can be compared with those predicted by FEM simulations, enabling the extraction of the material’s *E*, which is found to be 101$\:\pm\:$16 GPa. Figure 2g and **Supporting Table 1** collectively provide a summary of the measured *E* values for γ-GeSe, as determined by optical interferometry and AFM nano-indentation methods, alongside values obtained from density DFT calculations found in literature [33, 37, 50]. The experimentally measured *E* value was found to be consistent with those reported from the DFT calculations. Interestingly, γ-GeSe exhibits a higher *E* value compared to other group-IV monochalcogenides, whose *E* values typically range from 20 to 70 GPa [51–54]. 

**Fig. 3:**
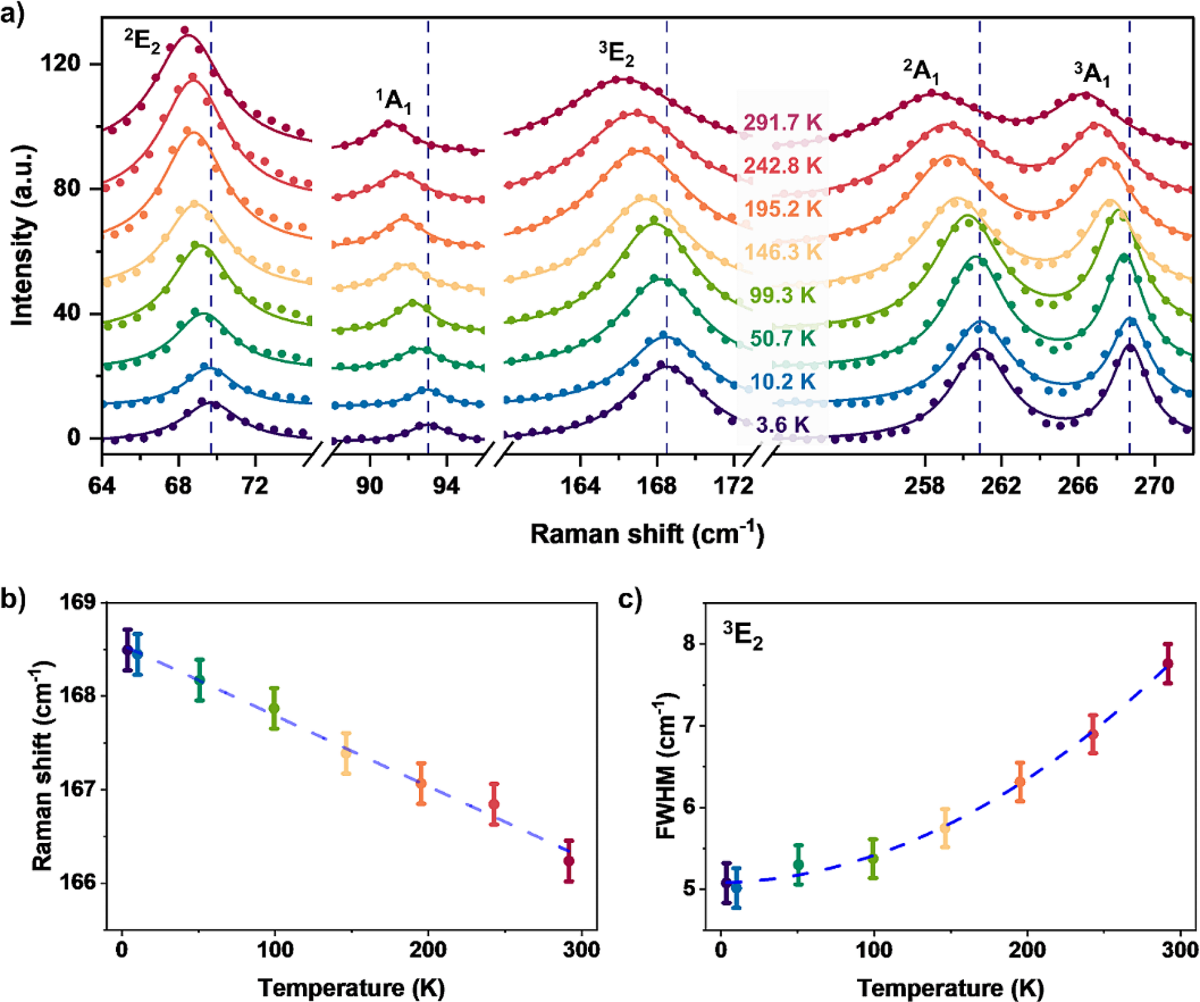
Temperature-dependent Raman spectroscopy of γ-GeSe. (**a**) Raman spectra of γ-GeSe on SiO_2_/Si substrate as a function of temperature. A red shift of Raman peaks at higher temperatures is apparent. (**b**) Temperature-dependent Raman shift and (**c**) full width half-maximum (FWHM) of ^3^E_2_ Raman mode

Optothermal Raman measurements, previously employed for determining the thermal conductivities of various nanomaterials, utilize temperature-dependent Raman signals to ascertain the local temperature of a sample under laser irradiation [55–57]. The thermal conductivity of the sample can be measured by applying heat transport equations. Initially, we conducted experiments to observe the temperature-dependent Raman modes of γ-GeSe supported by SiO_2_/Si substrate, ranging from 3.6 to 291.7 K, under vacuum conditions (Fig. 3). We identified five distinct Raman modes: ^2^E_2_ (69 cm^− 1^), ^3^E_2_ (168 cm^− 1^), ^1^A_1_ (93 cm^− 1^), ^2^A_1_ (261 cm^− 1^), and ^3^A_1_ (269 cm^− 1^), as shown in Fig. 3 and **Supporting Figure S3**. As the temperature increased, all Raman peaks of γ-GeSe exhibited red-shifting and broadening (Fig. 3b and c**)**. This behavior is consistent with that of other group-IV monochalcogenides, including α-GeSe, GeTe, SnSe, and SnTe [42, 58, 59]. In particular, the ^3^E_2_ mode is well isolated from the other Raman modes and displays a linear temperature-dependence up to room temperature with a first-order temperature coefficient of − 0.00757$\:\pm\:$0.00028 cm^−1^K^−1^. Therefore, the ^3^E_2_ mode was primarily utilized to monitor the sample’s local temperature for the following optothermal Raman measurements.

**Fig. 4:**
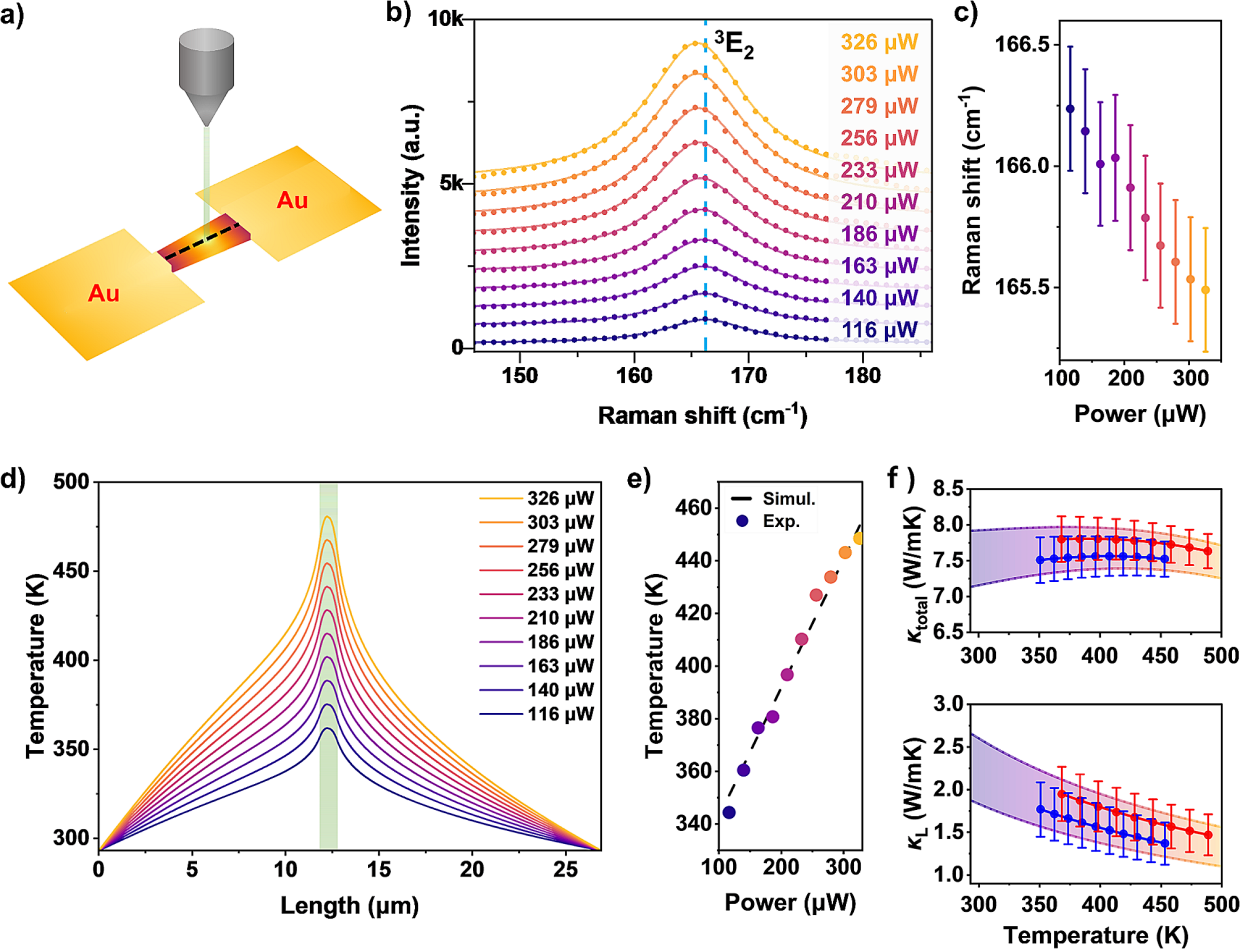
Optothermal Raman spectroscopy of γ-GeSe used in thermal conductivity measurements. (**a**) Schematic of the optothermal Raman spectroscopy measurement. (**b**) Raman spectra of a free-standing γ-GeSe as a function of the laser power. (**c**) Laser power-dependent Raman shift of ^3^E_2_. (**d**) Temperature profile simulation results of a freestanding γ-GeSe along the black line of the panel (**a**) for various laser power levels. The green area indicates the location under laser illumination. (**e**) Simulated and experimental temperature comparisons as a function of laser power. (**f**) Total (top) and lattice (bottom) thermal conductivities of γ-GeSe. Different colors correspond to measurements from different samples, and the error bars indicate the measurement uncertainty

We measured laser-induced local heating to investigate the thermal conductivity of γ-GeSe [60–62]. The focusing of laser beam (wavelength: 532 nm and ~ 0.5 μm focus size) onto the suspended region of the γ-GeSe sample induced heating of the sample (Fig. 4a). The laser-induced local heating measurements showed power-dependent, red-shifted Raman peaks (Fig. 4b and c and **Supporting Figure S4**). The Raman shift signal also exhibits a broad full-width at half maximum (FWHM), indicating heating of the sample. The observed equilibrium temperature profile along the suspended γ-GeSe is related to the thermal conductivity of γ-GeSe. By analyzing the local heating induced by laser irradiation and the temperature-dependent shifts in Raman peaks, we obtained the total thermal conductivity of γ-GeSe through comparison with the FEM simulation results. For a comprehensive understanding, a detailed description of the FEM simulation equations used in this study is provided in **Supporting Note 1**. Total thermal conductivity ($\:{\kappa\:}_{\text{t}\text{o}\text{t}\text{a}\text{l}}$) is the sum of the lattice thermal conductivity ($\:{\kappa\:}_{\text{L}}$) and electronic thermal conductivity ($\:{\kappa\:}_{\text{e}}$) [21]. We assumed that $\:{\kappa\:}_{\text{L}}$ is inversely proportional to temperature above the Debye temperature [32, 33, 63]. From previous measurement, γ-GeSe shows high *p*-type doping with $\:5\times\:{10}^{21}$ cm^− 3^ and $\:{\kappa\:}_{\text{e}}$ shows significant contribution to $\:{\kappa\:}_{\text{t}\text{o}\text{t}\text{a}\text{l}}$ [27]. We estimated the electronic thermal conductivity is $\:{\kappa\:}_{\text{e}}=\:$5.3 Wm^−1^K^−1^ at room temperature based on Wiedemann–Franz law with Lorenz number (L) of 2.44 × 10^−8^ V^2^K^−2^, which is consistent with the single parabolic band (SPB) model.

Figure 4d illustrates the equilibrium temperature profile along the suspended γ-GeSe flake under varying laser power conditions, as determined by the FEM simulations. By utilizing the first-order temperature coefficient of ^3^E_2_, we transformed the relationship between power and Raman shift into a temperature–laser power relationship (Fig. 4e and **Supporting Figure S5**). Figure 4e shows a good agreement between simulation and experiment. We measured a $\:{\kappa\:}_{\text{t}\text{o}\text{t}\text{a}\text{l}}$ of 7.5 $\:\pm\:$ 0.4 Wm^−1^K^−1^ along with a low $\:{\kappa\:}_{\text{L}}$ of 2.3$\:\pm\:$0.4 Wm^−1^K^−1^ (Fig. 4f and Supporting Table 2). The measured $\:{\kappa\:}_{\text{L}}$ value aligns with values reported in previous theoretical studies [31–35]. In particular, Minhas et al. reported a low $\:{\kappa\:}_{\text{L}}$ value of 1.73 Wm^− 1^K^− 1^ for bulk γ-GeSe [33]. Detailed calculations further suggest that such a low $\:{\kappa\:}_{\text{L}}$ could be attributed to the unique intralayer bonding configurations and the associated high anharmonicity in γ-GeSe. We note that the thermomechanical effect can be a source of error in thermal conductivity measurements based on optothermal Raman measurement and requires proper attention [64]. Although our analysis indicates that the thermomechanical effect is not significant in our experiment, it typically leads to an overestimation of the sample’s thermal conductivity. Therefore, proper consideration of this effect could result in an even lower thermal conductivity value of γ-GeSe.

**Fig. 5:**
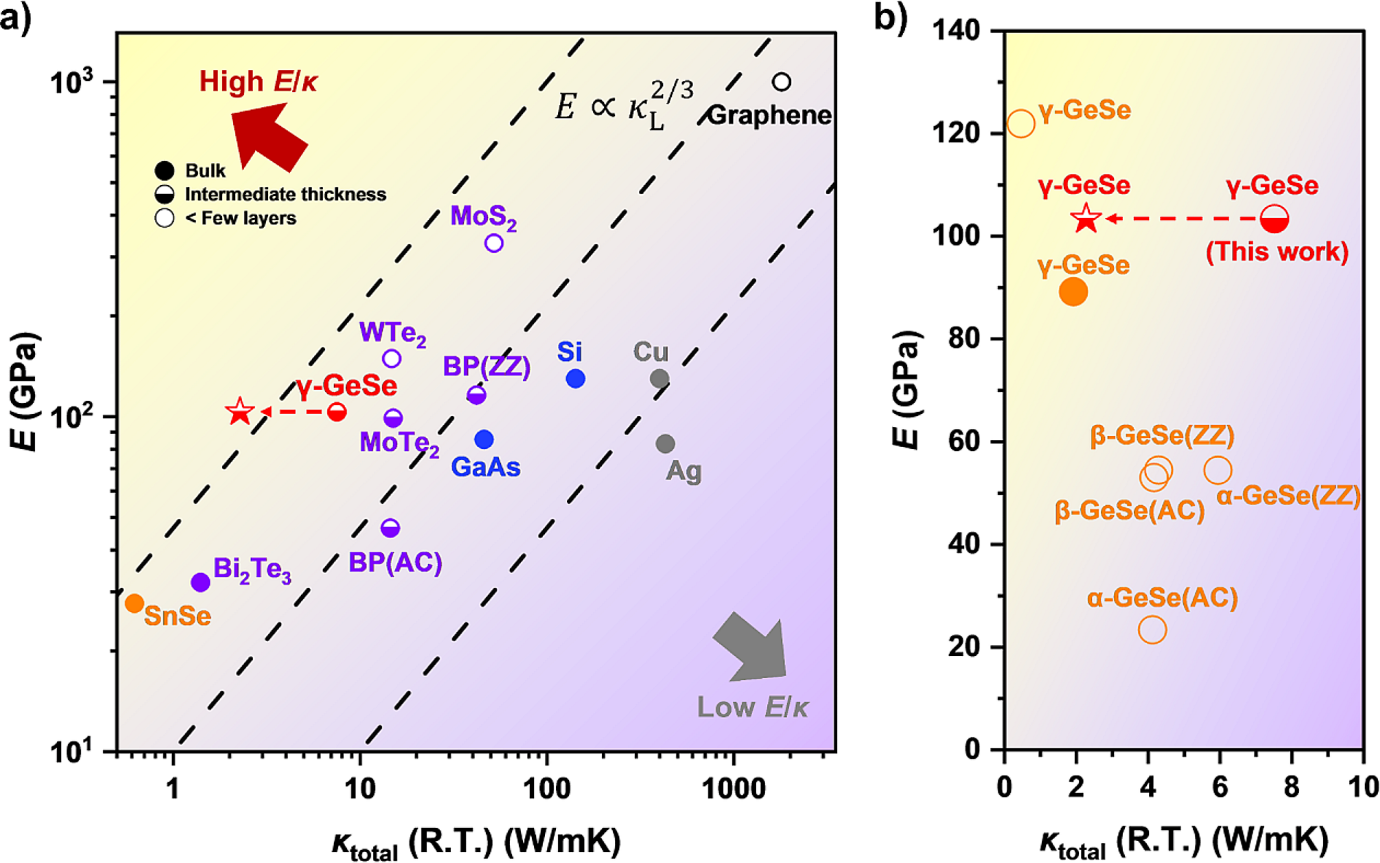
Thermal conductivity and Young’s modulus of various materials. (**a**) The values for γ-GeSe (red, this work), SnSe (orange), graphene (black), other 2D materials (purple), Si/GaAs (blue), and Cu/Ag (gray) are marked [39, 44, 47, 48, 54, 61, 62, 67–76]. For layered materials, the in-plane Young’s modulus (*E*) total thermal conductivity (*κ*_total_) are shown. The fully filled, half-filled, and open circles correspond to data from bulk samples, samples of intermediate thickness, and samples thinner than a few layers, respectively. The red star indicates the expected position of γ-GeSe without the $\:{\kappa\:}_{\text{e}}$ contribution, assuming a successful reduction in charge carrier concentration. (**b**) Thermal conductivity and Young’s modulus of α-, β-, and γ-GeSe. All data marked in orange represent calculation values based on DFT [34, 39, 77]. The data shown in red for γ-GeSe correspond to the experimentally measured value obtained in this work. The red star indicates the expected position of γ-GeSe without the $\:{\kappa\:}_{\text{e}}$ contribution, assuming a successful reduction in charge carrier concentration

Figure 5 shows the relationship between *E* and $\:{\kappa\:}_{\text{t}\text{o}\text{t}\text{a}\text{l}}$ for various types of materials. *E* is typically proportional to $\:\rho\:{\nu\:}_{\text{s}}^{2}$, where $\:\rho\:$ represents the density and $\:{\nu\:}_{\text{s}}$ is the sound velocity within a material. Conversely, $\:{\kappa\:}_{\text{L}}\:$is proportional to $\:{\nu\:}_{\text{s}}^{3}{T}^{-1}$ when the optical phonon contribution to the thermal conduction is minimal [34, 65]. Owing to these constraints, *E* and $\:{\kappa\:}_{\text{t}\text{o}\text{t}\text{a}\text{l}}$ show a positive correlation across a broad spectrum of materials. This relationship follows the proportionality $\:E\propto\:{\kappa\:}^{2/3},$ as depicted with the dashed lines in Fig. 5a.

γ-GeSe is located on the upper left side of Fig. 5a, indicating that it possesses a higher $\:E/{\kappa\:}_{\text{t}\text{o}\text{t}\text{a}\text{l}}$ ratio compared to other materials. This placement signifies the mechanical stability and low thermal conductivity of γ-GeSe. The $\:E/{\kappa\:}_{\text{t}\text{o}\text{t}\text{a}\text{l}}$ ratio is considered an important parameter for assessing the potential of materials in thermoelectric and related applications [66]. In comparison, metals (e.g., Cu, Ag) display a lower $\:E/{\kappa\:}_{\text{t}\text{o}\text{t}\text{a}\text{l}}$ ratio owing to their higher electrical conductivity and soft metal bonding characteristics. Moreover, two-dimensional (2D) layered-materials exhibit relatively higher $\:E/{\kappa\:}_{\text{t}\text{o}\text{t}\text{a}\text{l}}$ ratios compared to archetype semiconductors and metals. The relatively high $\:{E/\kappa\:}_{\text{L}}$ ratio identified in γ-GeSe is comparable to that of other promising thermoelectric materials such as SnSe and Bi_2_Se_3_. Figure 5b compares the in-plane $\:E$ and $\:{\kappa\:}_{\text{t}\text{o}\text{t}\text{a}\text{l}}$ among various types of GeSe polymorphs, highlighting the different mechanical and thermal properties of γ-GeSe compared to other GeSe polymorphs. Our results indicate that γ-GeSe exhibits a relatively high Young’s modulus compared to other GeSe polymorphs. We envision that doping engineering, specifically the reduction of doping concentration, could decrease the electronic contribution to thermal conductivity in γ-GeSe, thereby further increasing the $\:{E/\kappa\:}_{\text{L}}$ ratio, as illustrated in Fig. 5a and b.

## Conclusions

We investigated the mechanical and thermal properties of γ-GeSe using a freestanding sample geometry. Optical interferometry and nano-indentation using AFM revealed that the in-plane Young’s modulus (*E*) of γ-GeSe is 103.3$\:\pm\:$10.8 GPa, which is in alignment with the values obtained from DFT calculations reported in literature. Using the local heating induced by laser irradiation and the observed temperature-dependent Raman shifts, we were able to measure the in-plane thermal conductivity of γ-GeSe. This confirmed its low lattice thermal conductivity ($\:{\kappa\:}_{\text{L}}$) of 2.3$\:\pm\:$0.4 Wm^−1^K^−1^ at room temperature, a value that aligns well with predictions from DFT calculations. The low $\:{\kappa\:}_{\text{L}}$ of γ-GeSe can be attributed to its unique intralayer bonding configurations and the associated high anharmonicity. The relatively high ratio of $\:E/{\kappa\:}_{\text{L}}$ in γ-GeSe underscores the significant impact of a material’s bonding configuration on its mechanical and thermal properties.

## Methods

### Synthesis and characterizations

The γ-GeSe crystal was synthesized using CVD method [24]. γ-GeSe flakes were transferred onto a transmission electron microscopy (TEM) chip with a slit (Protochips Co., FIB-Optimized) utilizing a dry transfer method that involved the use of a polydimethylsiloxane (PDMS) support. Optical microscopy images were acquired using a Leica DM-750 M microscope with visible light. We used SEM (JSM-7001 F, JEOL) and AFM (XE7, Parksystems) to measure the geometry of the freestanding γ-GeSe samples.

### Optical interferometry

The mechanical resonance frequency was measured using an optical interferometry system under vacuum condition (10^− 6^ Torr). The power of He-Ne laser (633 nm) was below 0.5 mW. An AC signal was applied to the piezo ceramic substrate using a function generator (Tektronix AFG3102). Concurrently, a photodetector was used to detect the intensity of the laser beam reflected from the freestanding γ-GeSe. The detected signal was amplified using a lock-in amplifier (Stanford Research SR844).

### AFM nano-indentation

Nano-indentation was measured using an AFM (NX10, Parksystems) at the ambient condition. The spring constant of the AFM cantilever (RFESP-75, BRUKER) was accurately calibrated using a built-in thermal method and was set at 3 N/m (default).

### Raman spectroscopy

The temperature-dependent Raman spectra were recorded at temperatures ranging from 3.6 to 291.7 K using an optical cryostat (Montana instruments, s50). The measurements were carried out under vacuum (10^− 6^ Torr) with the 441.6 nm (2.81 eV) line of a He-Cd ion laser as the excitation source. The laser beam was focused onto the sample through a 40× objective lens (0.6 NA) in backscattering geometry. The laser power was kept below 50 µW to avoid degradation and local heating of the sample. The Raman signal was obtained using a Jobin-Yvon Horiba iHR550 spectrometer (2400 grooves/mm) combined with a liquid-nitrogen-cooled back-illuminated charge-coupled-device (CCD) detector. Volume holographic filters (OptiGrate) were utilized to block the Rayleigh-scattered light from entering the spectrometer.

### Laser-induced local heating

A Nd: YAG laser (532 nm) was directed onto the sample through a 100× objective lens and filtered by a D2 filter. The laser spot was carefully adjusted to achieve a radius of approximately 0.5 μm. The laser power was calibrated using a power meter. Raman signals were recorded employing a LabRam Aramis (HORIBA).

### Electronic supplementary material

Below is the link to the electronic supplementary material.

Supplementary Material 1

## Data Availability

The datasets used and/or analysed during the current study are available from the corresponding author on reasonable request.

## Abbreviations

vdW: Van der Waals
TMDC: Transition metal dichalcogenide
FEM: Finite element method
AFM: Atomic force microscopy
DFT: Density functional theory
CVD: Chemical vapor deposition
SEM: Scanning electron microscopy
SPB: Single parabolic band
2D: Two-dimensional
