# Supplementary material for: Unveiling the distinctive mechanical and thermal properties of γ-GeSe
Source: Nano Converg. 2024 Jul 15;11:29. doi: 10.1186/s40580-024-00436-3 (PMC11250563; doi:10.1186/s40580-024-00436-3)
Supplement: Supplementary file 1 — Supplementary Material 1 [file 40580_2024_436_MOESM1_ESM.docx]

Supplementary Information

**Unveiling the Distinctive Mechanical and Thermal Properties of γ-GeSe**

Jinsub Park^1†^, Yugyeong Je^2†^, Joonho Kim^1^, Je Myoung Park^3^, Joong-Eon Jung^1^, Hyeonsik Cheong^3^, Sang Wook Lee^2*^, and Kwanpyo Kim^1*^

^1^Department of Physics, Yonsei University, Seoul 03722, Republic of Korea

^2^Department of Physics, Ewha Womans University, Seoul 03760, Republic of Korea

^3^Department of Physics, Sogang University, Seoul 04107, Republic of Korea

^†^Jinsub Park and Yugyeong Je contributed equally to this work.

^*^Corresponding authors: Sang Wook Lee, Kwanpyo Kim

E-mail: S. W. L. ([leesw@ewha.ac.kr](mailto:leesw@ewha.ac.kr)), K. K. ([kpkim@yonsei.ac.kr](mailto:kpkim@yonsei.ac.kr))

Supporting Note 1. Finite element method (FEM) simulations

We used the structural mechanics and heat transfer modules of COMSOL Multiphysics software for investigating mechanical and thermal properties of γ-GeSe. The irregular sample geometry (varying length, width, and thickness) measured by scanning electron microscopy (SEM) and atomic force microscopy (AFM) was incorporated into simulations, as shown in **Supporting Figure S2**.

(a) Mechanical properties

For experimental measurements of mechanical properties of γ-GeSe, we acquired the mechanical resonances in out-of-plane vibration and out-of-plane deflection using freestanding structure. These properties are determined by Young’s modulus, Poisson’s ratio, and shear modulus. We used the linear elastic orthotropic model for FEM simulation. The equations for the simulations are expressed as

$\rho\omega^{2}\boldsymbol{u+\nabla\cdot\sigma=0;}\omega\mathbf{=}2\pi f$; $\mathbf{u}=\mathbf{u}_{0}e^{\left( i\omega t \right)}$ (1-1)

$\boldsymbol{\nabla}\cdot\boldsymbol{\sigma}+\mathbf{F}=\rho\ddot{\mathbf{u}}$ (1-2)

$\boldsymbol{\epsilon}=\frac{1}{2}\left[ \boldsymbol{\nabla u}+\left( \boldsymbol{\nabla u} \right)^{T} \right]$ (2)

$\boldsymbol{\sigma}=C\boldsymbol{:}\boldsymbol{\epsilon}$ (3)

$C^{-1}=\left[ \begin{matrix} \begin{matrix} \frac{1}{E_{x}} & -\frac{\nu_{\mathrm{yx}}}{E_{y}} & -\frac{\nu_{\mathrm{zx}}}{E_{z}} & 0 & 0 & 0 \\ -\frac{\nu_{xy}}{E_{y}} & \frac{1}{E_{y}} & -\frac{\nu_{\mathrm{zy}}}{E_{z}} & 0 & 0 & 0 \\ -\frac{\nu_{\mathrm{xz}}}{E_{x}} & -\frac{\nu_{\mathrm{yz}}}{E_{y}} & \frac{1}{E_{z}} & 0 & 0 & 0 \\ 0 & 0 & 0 & \frac{1}{G_{\mathrm{yz}}} & 0 & 0 \\ 0 & 0 & 0 & 0 & \frac{1}{G_{\mathrm{xz}}} & 0 \\ 0 & 0 & 0 & 0 & 0 & \frac{1}{G_{\mathrm{xy}}} \end{matrix} \end{matrix} \right]$ (4)

$\nu_{\mathrm{ij}}E_{j}=\nu_{\mathrm{ji}}E_{i}$; $\frac{\nu_{\mathrm{ij}}}{\nu_{\mathrm{ji}}}=\frac{E_{i}}{E_{j}}$ (5)

$G_{\mathrm{ij}}=\frac{E_{i}E_{j}}{E_{i}+E_{i}+2E_{i}\nu_{\mathrm{ij}}}$ (6)

$E_{\mathrm{IP}}=E_{x}=E_{y}$; $\nu_{\mathrm{xy}}=\nu_{\mathrm{yx}}$ (7)

where $\rho$ is the density of γ-GeSe (5.43 g/cm^3^), **F** is the body force unit per volume, **u** is the displacement vector, $f$ is the eigenfrequency, and $C$ is the stiffness tensor. Originated from the hexagonal symmetry, γ-GeSe has isotropic in-plane mechanical properties as shown in **Equation (7)**.

The experimental measurement is mainly determined by in-plane Young’s modulus ($E_{\mathrm{IP}}$) and the out-of-plane Young’s modulus ($E_{\mathrm{OOP}}$) shows no significant effect. To confirm that $E_{\mathrm{OOP}}$ is mostly irrelevant, we simulated mechanical resonance and indentation distance as a function of $E_{\mathrm{OOP}}\text{/}E_{\mathrm{IP}}$ ratio ranging from 0.1 to 10.[1] Our simulation confirmed that $E_{\mathrm{OOP}}$ is not relevant to mechanical resonance frequency and nano-indentation deflection as shown in **Supporting Figure S6a and S6b**. In-plane Poisson ratio ($\nu_{xy}$) of 0.254 was adopted from the recent calculation results.[2–4]

**(b) Thermal properties**

For the measurement of thermal properties of γ-GeSe, we acquired the temperature change via optothermal Raman experiment. The equations for the heat transfer are expressed as

$\boldsymbol{\nabla\cdot}\mathbf{q}=Q$ (8)

$\mathbf{q}=-\kappa_{\mathrm{total}} \nabla T$ (9)

$\kappa_{\mathrm{total}}=\kappa_{L}+\kappa_{e}$ (10)

$\kappa_{L}=\frac{\alpha}{T}$ (11)

where $\mathbf{q}$ is the heat flux, $Q$ is heat input power from the laser irradiation, $\kappa_{\mathrm{total}}$ is total thermal conductivity, and T is temperature. We assumed that the laser was uniformly absorbed at surface of γ-GeSe in the circular area with 0.5 μm diameter. The laser absorbance *A* of the sample was measured from optical reflectance (*R*) and transmittance (*T*) of the samples near the wavelength of laser, with $A=1-T-R$, as shown in **Supporting Figure S7**. The clamping regions of the suspended sample serve as a perfect heat sink and fixed at room temperatures. In our experiments, gold deposition of thickness 100 nm at clamping ensures the facilitated heat transport. We assumed that the lattice thermal conductivity ($\kappa_{L}$) is inversely proportional to temperature T. Electronic thermal conductivity ($\kappa_{e}$) is estimated from Wiedemann–Franz law and sample’s electrical conductivity. We extracted $\alpha$ and $\kappa_{L}$ by minimization of the 2^nd^ norm errors compared with experimental results.

Due to the experimental geometry, the in-plane total thermal conductivity ($\kappa_{\mathrm{IP}}$) is mainly relevant to our measurements and the out-of-plane thermal conductivity ($\kappa_{\mathrm{OOP}}$) is irrelevant. We performed the laser-induced heating simulation as a function of $\kappa_{\mathrm{OOP}}/\kappa_{\mathrm{IP}}$ as show in **Supporting Figure S6c**. We confirmed that the out-of-plane total thermal conductivity ($\kappa_{\mathrm{OOP}}$) have negligible effect on our measurements.

| **Sample number** | **Thickness (nm)** | | **Trench  Length (μm)** | **Young’s modulus (GPa)** | |
| --- | --- | --- | --- | --- | --- |
|  | **Thin** | **Thick** |  | **Optical interferometry** | **AFM nano-indentation** |
| **1** | **300** | **390** | **20** | **106** | **125**$\boldsymbol{\pm}$**4** |
| **2** | **300** | **330** | **20** | **93** | **102**$\boldsymbol{\pm5}$ |
| **3** | **60** | **260** | **21** | **93** | **---** |
| **4** | **220** | **420** | **25** | **---** | **101**$\boldsymbol{\pm}$**16** |

**Supporting Table1. Measured in-plane Young’s modulus (**$\boldsymbol{E}_{\mathbf{IP}}$**) of γ-GeSe.**

| **Sample number** | **Thickness (nm)** | | **Trench  Length (μm)** | $\boldsymbol{\kappa}_{\mathbf{total}}$  **(W/mK)** | $\boldsymbol{\kappa}_{\mathbf{L}}$  **(W/mK)** |
| --- | --- | --- | --- | --- | --- |
|  | **Thin** | **Thick** |  |  |  |
| **5** | **100** | **300** | **20** | **7.4**$\boldsymbol{\pm}$**0.4** | **2.1**$\boldsymbol{\pm}$**0.4** |
| **6** | **120** | **150** | **22** | **7.7**$\boldsymbol{\pm}$**0.4** | **2.4**$\boldsymbol{\pm}$**0.4** |

**Supporting Table2. Total and lattice in-plane thermal conductivity of γ-GeSe.**


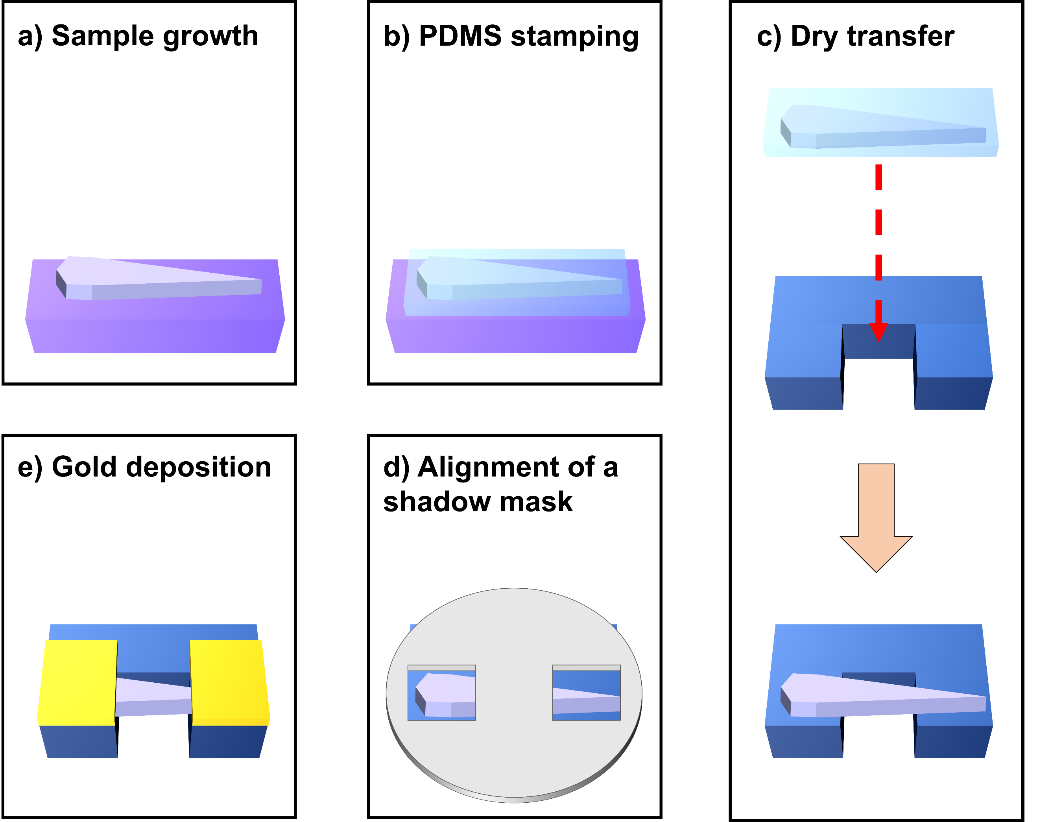


**Supporting Figure S1. Sample fabrication procedure of doubly-clamped freestanding γ-GeSe.** (a) γ-GeSe growth on gold-coated SiO_2_/Si substrate. (b) Stamping of PDMS film to a target γ-GeSe sample. (c) Dry transfer of γ-GeSe to a trenched substrate. (d) Alignment of a metal shadow mask on γ-GeSe for metal coating near clamping regions. (e) Gold deposition (100 nm) for clamping.


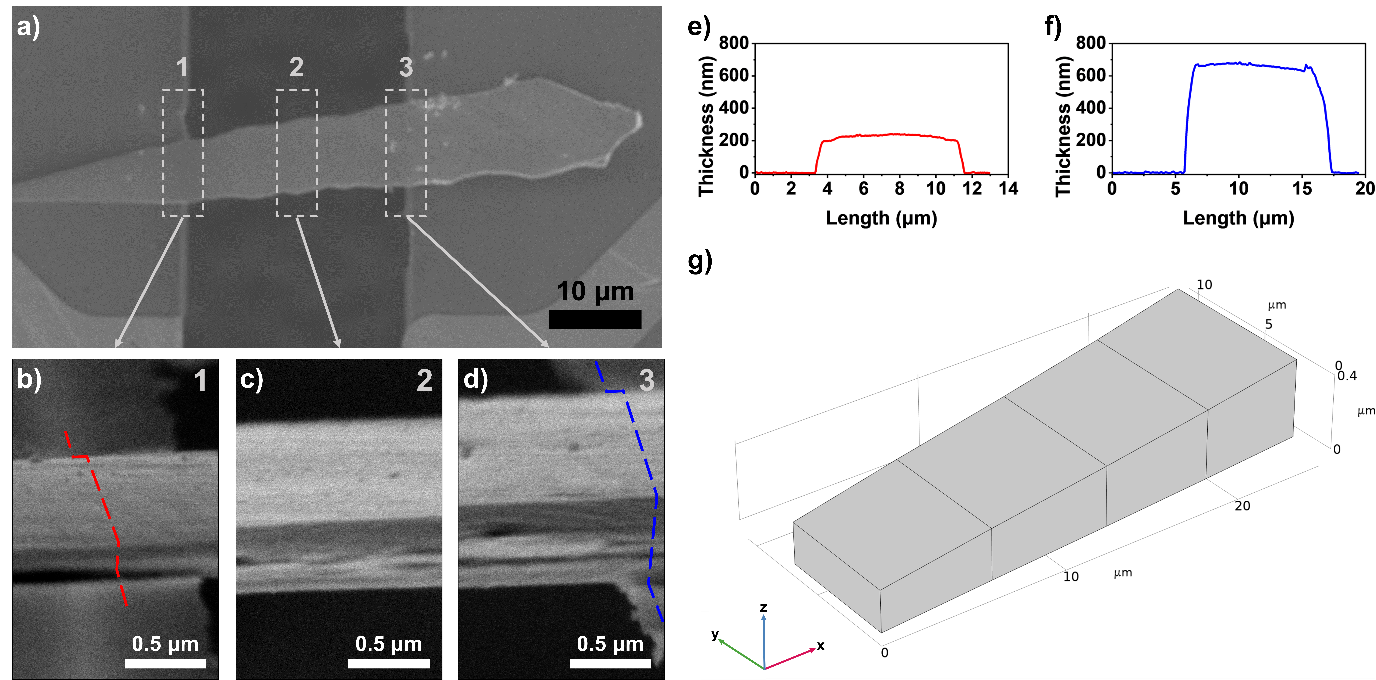


Supporting Figure S2. Experimental confirmation of thickness and width in suspended γ-GeSe samples. The thickness of each freestanding γ-GeSe samples were measured using SEM and AFM imaging. (a) SEM image of a sample from top-view. (b-d) Thickness measurement from SEM side-view, focusing on different sections (positions 1, 2, and 3, respectively). These images were captured at an 85° tilt from the top viewing direction. (e-f) AFM line profiles along the red (b) and the blue (d) dashed-lines, respectively. (g) FEM simulation geometry of the freestanding region.


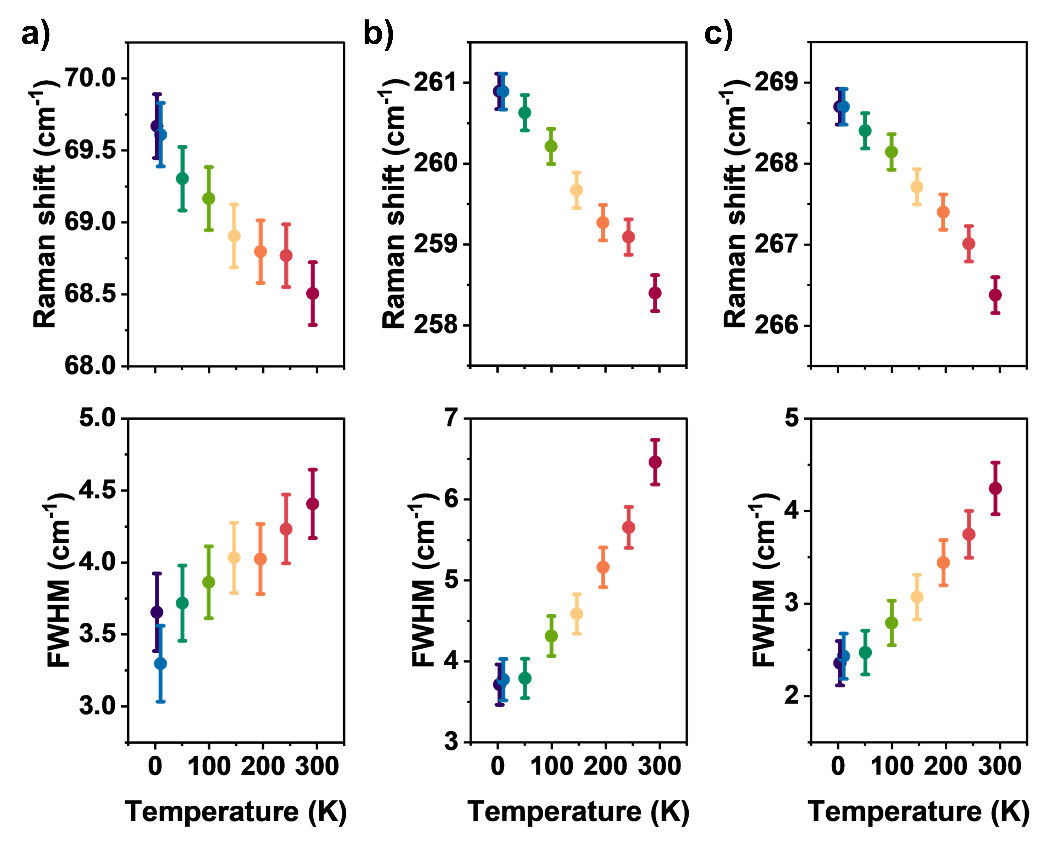


**Supporting Figure S3. Temperature-dependent Raman shifts and full width half-maximum (FWHM).** The data for (a) ^2^E_2_, (b) ^2^A_1_, and (c) ^3^A_1_ are presented.


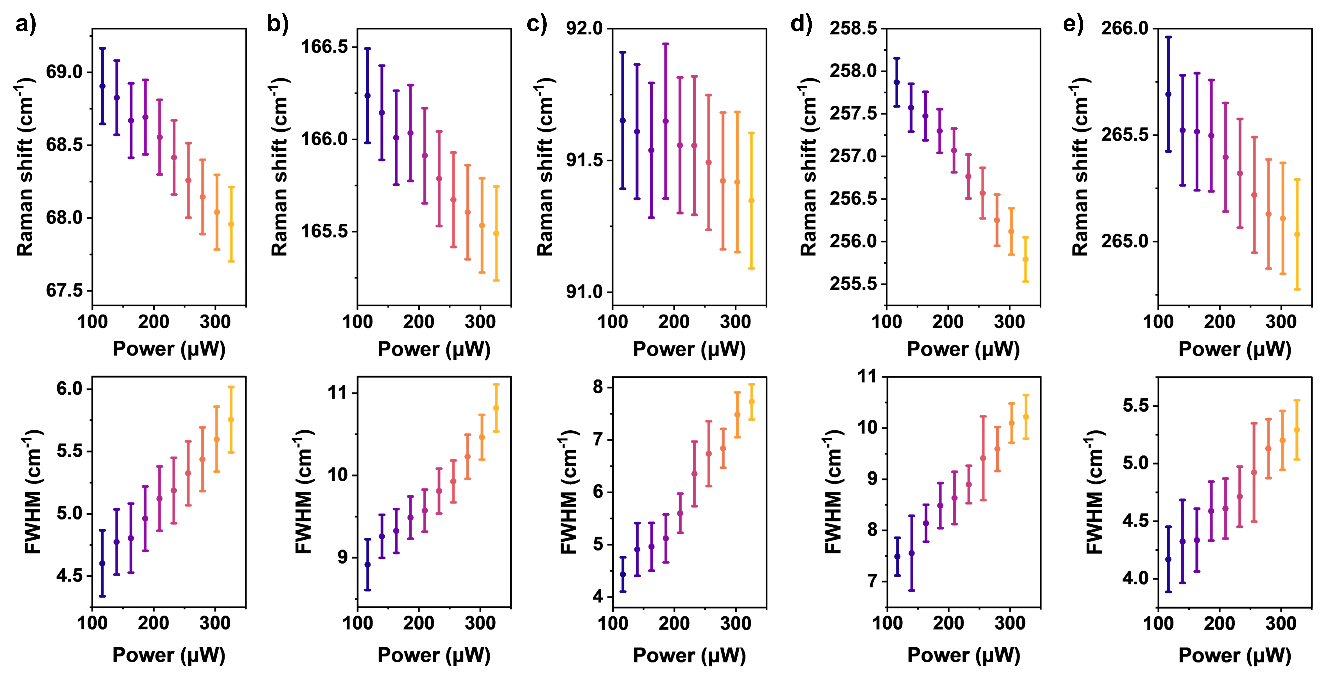


**Supporting Figure S4. Laser power-dependent Raman shift and FWHM of sample.** (a)-(e) Analysis of ^2^E_2_, ^3^E_2_, ^1^A_1_, ^2^A_1_, and ^3^A_1_ modes under varying laser power. Error bars represent the standard deviation from three repeated experiments.


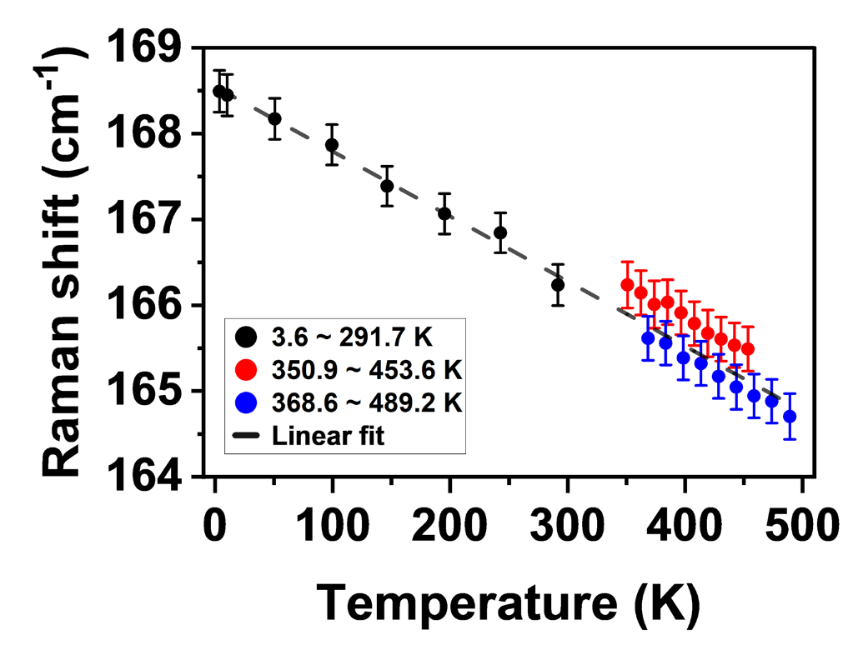


**Supporting Figure S5. Temperature-dependent Raman shift of ^3^E_2_.** (a) The laser power-dependent Raman shift of ^3^E_2_ was converted the local temperature using the first-order thermal expansion coefficient of ^3^E_2_. The total thermal conductivity of γ-GeSe was calculated based on FEM simulations and the observed power dependent temperature changes.


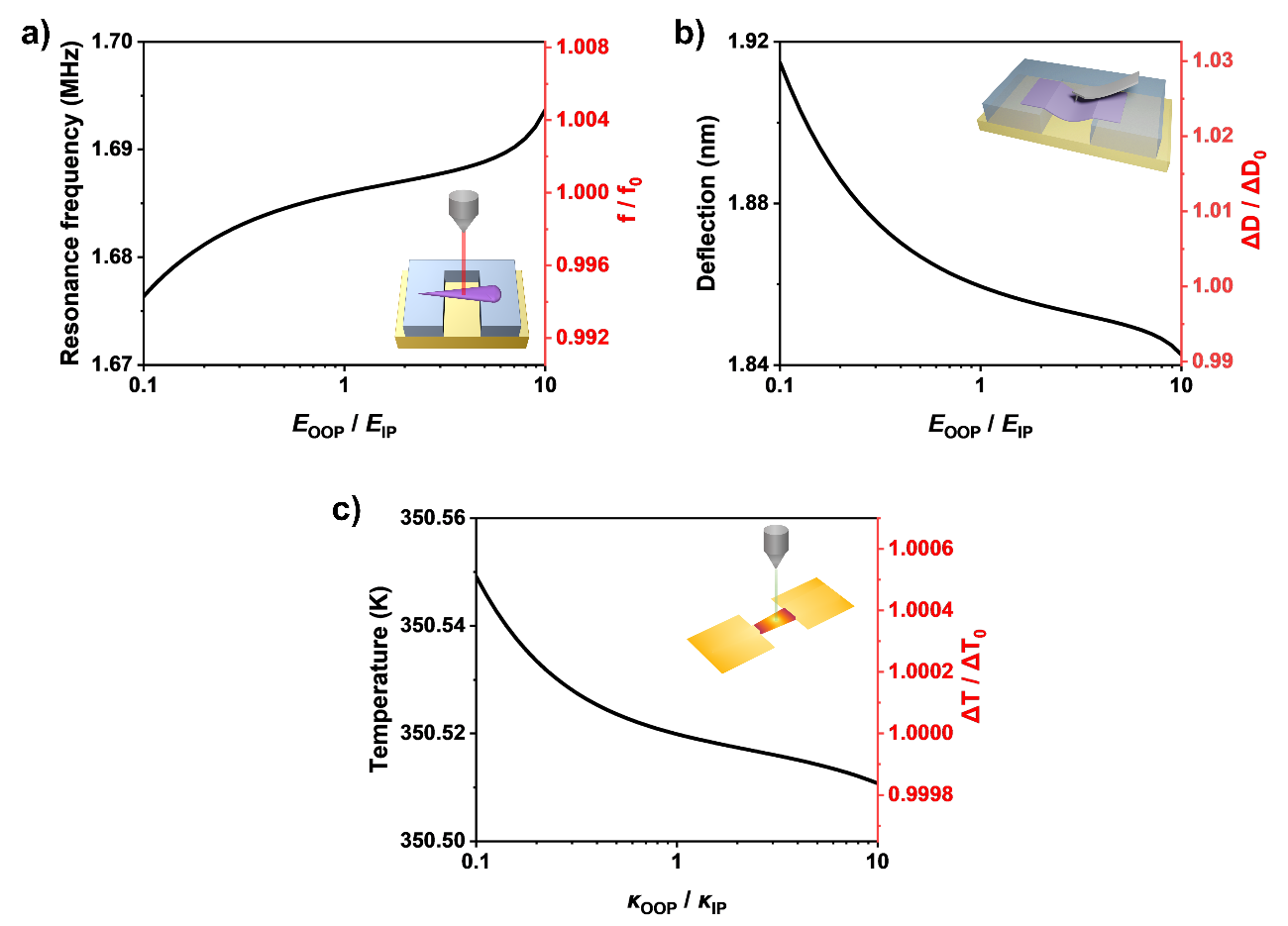


**Supporting Figure S6. Simulation on the effect of out-of-plane Young’s modulus (**$\boldsymbol{E}_{\mathbf{OOP}}$**) and out-of-plane thermal conductivity (**$\boldsymbol{\kappa}_{\mathbf{OOP}}$**).** (a) Simulated mechanical resonance frequency as a function of $E_{\mathrm{OOP}}\text{/}E_{\mathrm{IP}}$ ratio. $f_{0}$ is the resonance frequency with $E_{\mathrm{OOP}}\text{/}E_{\mathrm{IP}}=1$. (b) Simulated indentation distance as a function of $E_{\mathrm{OOP}}\text{/}E_{\mathrm{IP}}$ ratio. $D_{0}$ is the deflection at the location of X = 10 μm with $E_{\mathrm{OOP}}\text{/}E_{\mathrm{IP}}=1$. (c) Simulated temperature at the location under the laser illumination at power of 116 μW as a function of $\kappa_{\mathrm{OOP}}/\kappa_{\mathrm{IP}}$ ratio.


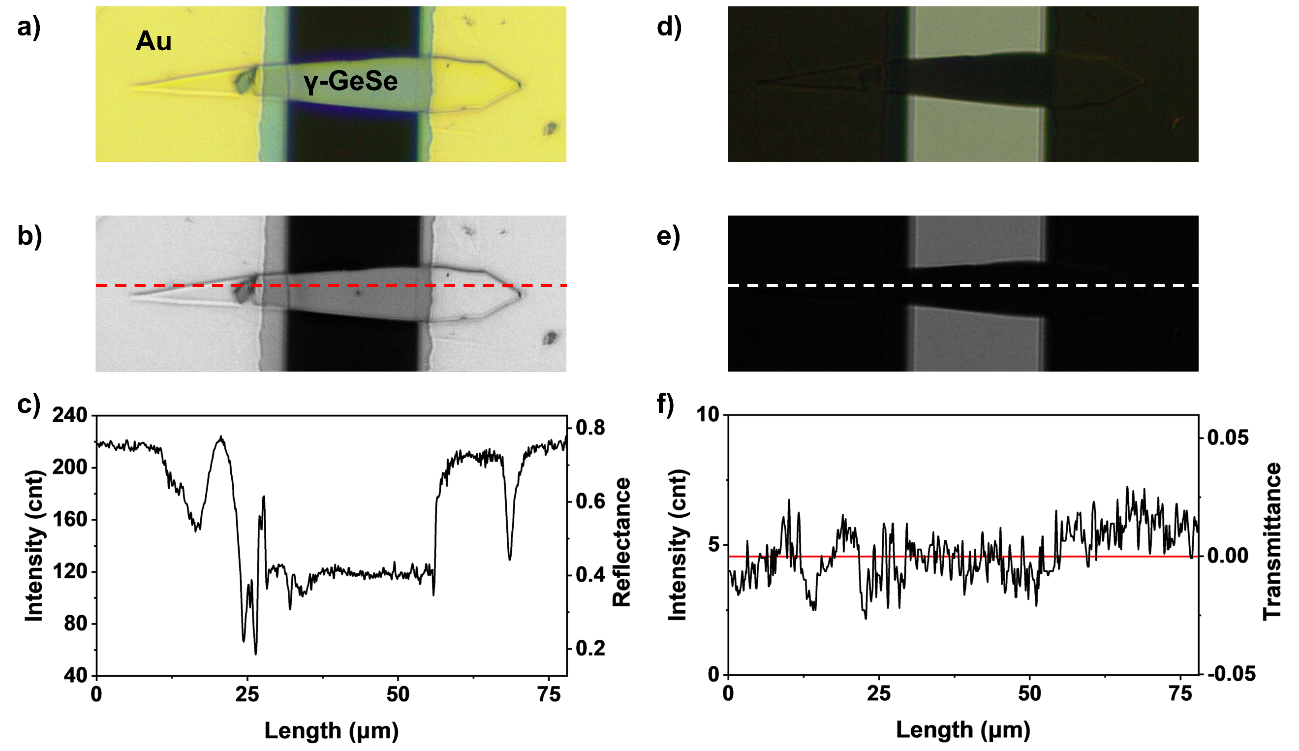


**Supporting Figure S7. Determination of laser absorbance onto γ-GeSe samples.** The laser absorbance *A* of the sample was measured from optical reflectance (*R*) and transmittance (*T*) of the samples near the wavelength of laser, with $A=1-T-R$. (a) Optical image of a suspended γ-GeSe sample under reflection mode. (b) Gamma-corrected optical image in the green-split channel. (c) Pixel intensity and corresponding reflectance of red-dashed line in panel b. Reflectance of gold is adapted from Sopra material database. (d) Optical image of a suspended γ-GeSe sample under transmission mode. (e) Gamma-corrected optical image in the green-split channel. (f) Pixel intensity and corresponding transmittance of white-dashed line in panel e.

**Supporting References**

1. S. Manna, P. Gorai, G. L. Brennecka, C. V. Ciobanu, and V. Stevanović, J. Mater. Chem. **6**, 11035 (2018).

2. H. Minhas, S. Das, and B. Pathak, ACS Appl. Energy Mater. **5**, 9914 (2022).

3. C. Huan, P. Wang, B. He, Y. Cai, and Q. Ke, 2D Mater. **9**, 045014 (2022).

4. V. Van Thanh, N. D. Van, D. Van Truong, and N. T. Hung, Appl. Surf. Sci. **582**, 152321 (2022).
